# BMP4 signaling is involved in the generation of inner ear sensory epithelia

**DOI:** 10.1186/1471-213X-5-16

**Published:** 2005-08-17

**Authors:** Huawei Li, Carleton E Corrales, Zhengmin Wang, Yanling Zhao, Yucheng Wang, Hong Liu, Stefan Heller

**Affiliations:** 1 Department of Otolaryngology and Program in Neuroscience, Harvard Medical School and Eaton Peabody Laboratory, Massachusetts Eye and Ear Infirmary, Boston, MA 02114, USA; 2 Department of Otolaryngology, Central Laboratory of Eye, Ear, Throat and Nose Hospital, Shanghai Medical College of Fudan University, Shanghai, 200031, PR of China

## Abstract

**Background:**

The robust expression of BMP4 in the incipient sensory organs of the inner ear suggests possible roles for this signaling protein during induction and development of auditory and vestibular sensory epithelia. Homozygous BMP4-/- animals die before the inner ear's sensory organs develop, which precludes determining the role of BMP4 in these organs with simple gene knockout experiments.

**Results:**

Here we use a chicken otocyst culture system to perform quantitative studies on the development of inner ear cell types and show that hair cell and supporting cell generation is remarkably reduced when BMP signaling is blocked, either with its antagonist noggin or by using soluble BMP receptors. Conversely, we observed an increase in the number of hair cells when cultured otocysts were treated with exogenous BMP4. BMP4 treatment additionally prompted down-regulation of Pax-2 protein in proliferating sensory epithelial progenitors, leading to reduced progenitor cell proliferation.

**Conclusion:**

Our results implicate BMP4 in two events during chicken inner ear sensory epithelium formation: first, in inducing the switch from proliferative sensory epithelium progenitors to differentiating epithelial cells and secondly, in promoting the differentiation of hair cells within the developing sensory epithelia.

## Background

The inner ear is derived from a thickened patch of ectodermal cells, the otic placode, which develops lateral to the developing hindbrain. In birds and mammals, the otic placode invaginates and pinches off to form the pear-shaped otic vesicle, or otocyst. The inner ear's sensory epithelia originate from the ventromedial region of the otocyst, an area that can be defined by the expression of several markers, such as BEN (bursal epithelium and neurons)[1], lunatic fringe and cSerrate-1 [2-4], islet-1 [5] and bone morphogentic proteins (BMPs) [4,6,7]. The distinctive temporal and spatial expression patterns of BMP4 in almost all developing chicken inner ear sensory epithelia has led to the suggestion that this protein may play an important role in the induction of inner ear sensory organs [4,6]. Because homozygous BMP4 knockout mice die between E6.5 and E9.5 [8], a period before the inner ear has formed, it is difficult to analyze the role of BMP4 in the genesis of inner ear sensory organs. As a consequence, other model systems, such as the chicken embryo, have been used to study possible roles of BMP4 in the inner ear. For example, noggin, an antagonist of BMPs, has been employed to interfere with BMP signaling during chicken inner ear development resulting in defects in semicircular canal formation and otic capsule malformation [9,10]. Although malformed or missing cristae were observed in these experiments, the hair cells developed normally. Thus these results did not clarify why BMP4 is robustly expressed in sensory epithelia primordia thereby neither confirming nor refuting the hypothesis that BMP signaling is involved in the genesis of inner ear sensory organs. A possible explanation for the previously observed lack of sensory epithelia defects after *in ovo *application of BMP4 antagonists is that the antagonists did not penetrate far enough to reach sufficiently high concentrations in the developing sensory epithelia to block BMP signaling effectively. To address this issue, we exploited a serum-free floating otocyst culture system, which allowed us to quantitatively analyze progenitor cell proliferation, apoptosis and cell differentiation in the developing otocyst with loss of function and gain of function experiments. Our experiments revealed that BMP4 signaling is involved in generation of sensory epithelia by negatively regulating inner ear progenitor cell proliferation through downregulation of the homeodomain transcription factor Pax-2. Subsequently, BMP4 signaling promotes hair cell differentiation.

## Results

### Exogenous BMP4 leads to increase in hair cell numbers and blockade of BMP signaling inhibits hair cell generation

**Figure 1:**
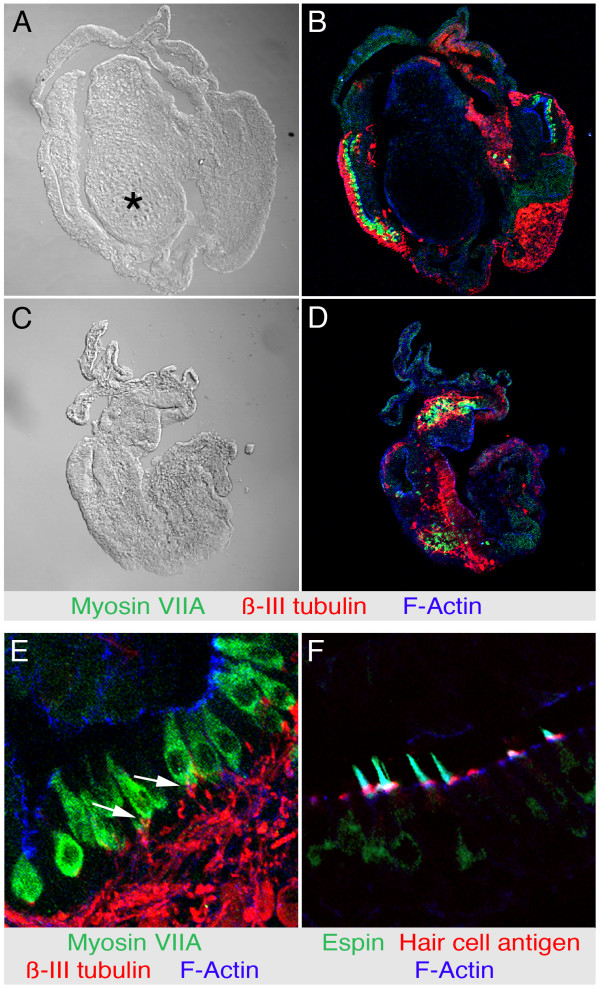
Stage 16 otocysts cultured for 7 days develop hair cells and neurons, independent of the presence of mesenchymal tissue. *(A) *Incomplete removal of periotic mesenchyme is clearly visible in cryosections of otocysts cultured for seven days. The asterisk labels such an area of mesenchyme-derived cells with chondrocyte morphology that label poorly with phalloidin (shown in B), indicating lower levels of F-actin in mesenchymal derivatives. *(B) *Hair cells, visualized by green myosin VIIA immunofluorescence and neurons (red β-III tubulin immunofluorescence) develop in the presence of mesenchyme. Filamentous actin is visualized with phalloidin-labeling (blue fluorescence) *(C) *Mesenchyme-free otocysts do not display mesenchyme-derived cell morphologies. *(D) *Hair cells (green) and neurons (red) develop in mesenchyme-free otocysts. Filamentous actin is visualized with phalloidin-labeling (blue fluorescence). *(E) *β-III tubulin-positive neurites (red) appear to contact myosin VIIA-positive hair cells shown in green (arrows). *(F) *Hair cells develop hair bundles that can be visualized with antibody to espin (green fluorescence), hair cell antigen (red fluorescence), and filamentous actin (blue fluorescence).

Maintenance of the developing avian inner ear *in vitro *in an environment with largely reduced extrinsic influences can be facilitated by removal of periotic mesenchyme, which otherwise could serve as source for various signaling molecules [11,12]. As much as possible, we assured removal of the mesenchymal tissue surrounding the otocyst by mild enzymatic treatment with trypsin and careful dissection. We found that removal of the periotic mesenchyme did not affect the capability of stage 16 otic vesicles to generate major inner ear cell types after 7d in serum-free suspension culture (Fig. 1). Vesicles with obvious mesenchymal contributions that gave rise to cells with chondrocyte morphology (Fig. 1A, B) were excluded from this study. Only otic vesicles that did not display any obvious sign of mesenchymal contribution after culture were included in this study (Fig. 1C, D).

Although free-floating otocysts did not differentiate into morphologically well-defined and structured inner ears, they displayed remarkable constancy with regard to the number of total cells after 7 days in culture (approximately 40,000 cells per otocyst; Mutai and Heller, unpublished data) and of the number of hair cells formed during the culture period (648 ± 117 hair cells per otocyst, n = 8; about 1.6% of the total cell number). We identified hair cells with antibody to myosinVIIA [13] and by their hair bundles labeled with antibody to the hair bundle markers espin and hair cell antigen [14-16] (Fig. 1E, F). Furthermore, the hair cells that differentiated in free-floating otocysts appeared to be contacted by neurites, identified with antibody to neuron-specific β-III tubulin (Fig. 1E).

**Figure 2:**
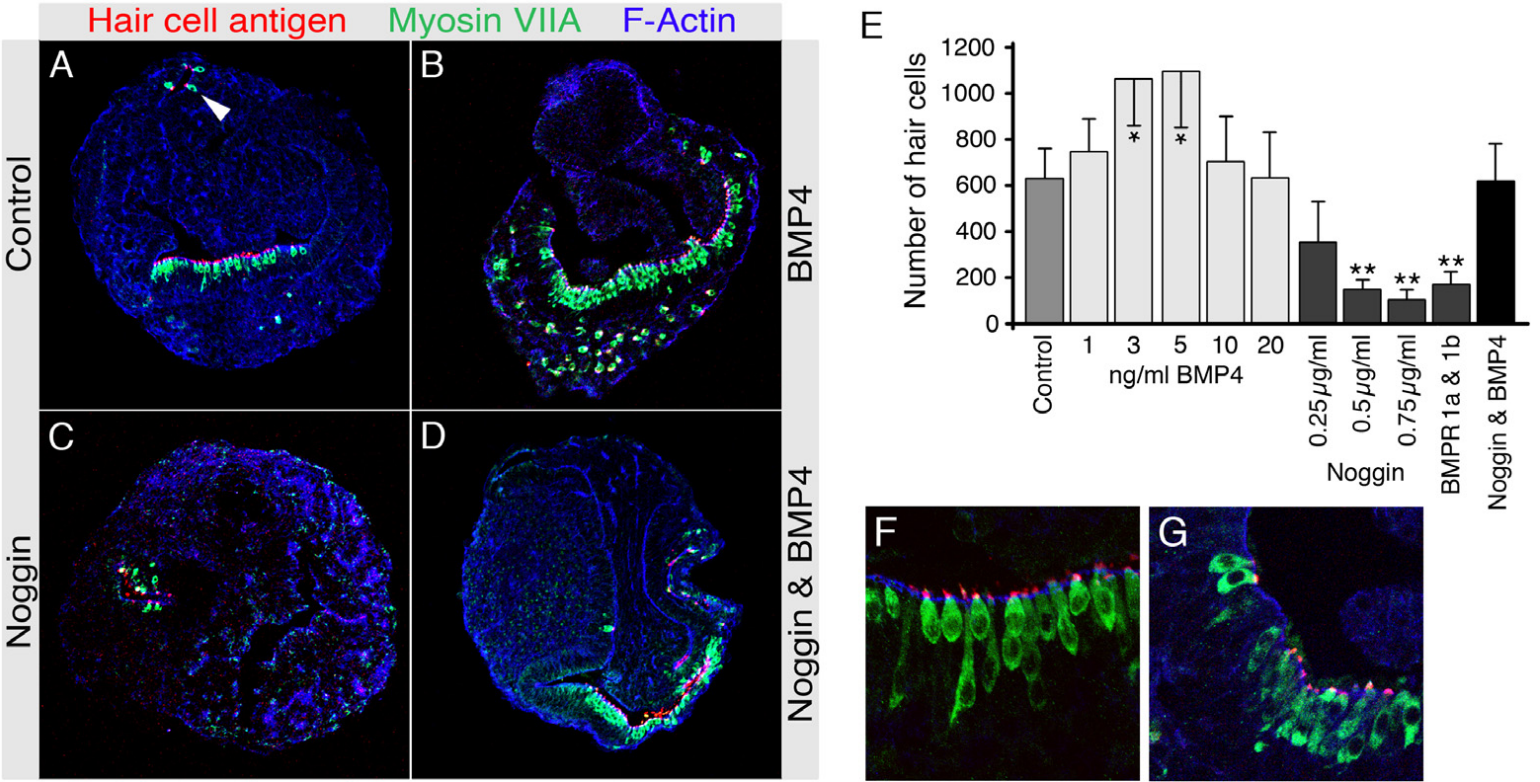
Increased production of hair cells with exogenous BMP4 and decrease in hair cell numbers in response to blockade of BMP signaling. *(A) *Hair cell antigen (red) and myosin VIIA-positive hair cells (green) in a cryosectioned otocyst after seven days culture. Filamentous actin is visualized with phalloidin-labeling (blue fluorescence). We routinely observed both hair cells organized in epithelia (see also higher magnification in (F)) and scattered hair cells (arrowhead). *(B) *BMP4 at 5 ng/ml, applied on the third day in culture, leads to substantial increase in the number of hair cells in epithelial cells and also in the scattered population of isolated hair cells. *(C) *Noggin at 0.5 μg/ml diminishes the number of hair cells. *(D) *The effect of noggin-treatment (0.5 μg/ml)) can be rescued by addition of 5 ng/ml exogenous BMP4. *(E) *Dose-dependence of the effect of exogenously added BMP4 on the number of hair cells in otocysts after seven days in culture. BMP4 at 3 ng/ml and at 5 ng/ml significantly increases the number of hair cells when compared with control conditions (asterisks indicate *p *< 0.05, unpaired Student's t-test, n = 4–5). Noggin at various concentrations and soluble BMPR 1a and 1b significantly reduced the number of hair cells detected in otocysts after seven days in culture when compared to the untreated control (asterisks indicate *p *< 0.003, unpaired Student's t-test, n = 6–7); the effect of 0.5 μg/ml noggin can be fully rescued by addition of 5 ng/ml BMP4. Error bars represent standard deviations. *(F,G) *Higher magnification to show the morphology of hair cells observed in (A) and in (B).

To assess the effects of BMP signaling on otocyst cells and hair cell generation, we analyzed complete sets of serial sections obtained from individual specimens of each experimental group. When exogenous BMP4 was added on culture day three, we observed a substantial increase in the number of hair cells, when compared with the untreated controls (Fig. 2A, B, F, G); hair cells were identified by double-immunostaining for myosinVIIA and hair cell antigen. Blocking of BMP signaling with either noggin or soluble BMP receptor 1a or 1b proteins dramatically reduced hair cell numbers after seven days in culture (Fig. 2C, E). The effect of noggin on hair cell generation was dosage-dependant and could be rescued by adding exogenous BMP4 (Fig. 2D, E). Exposing cultures to different concentrations of BMP4 revealed an optimal concentration of 3–5 ng/ml, which yielded the greatest number of hair cells (Fig, 2E).

### BMP4 inhibits otocyst cell proliferation and induces apoptosis in prospective inner ear ganglion cells

**Figure 3:**
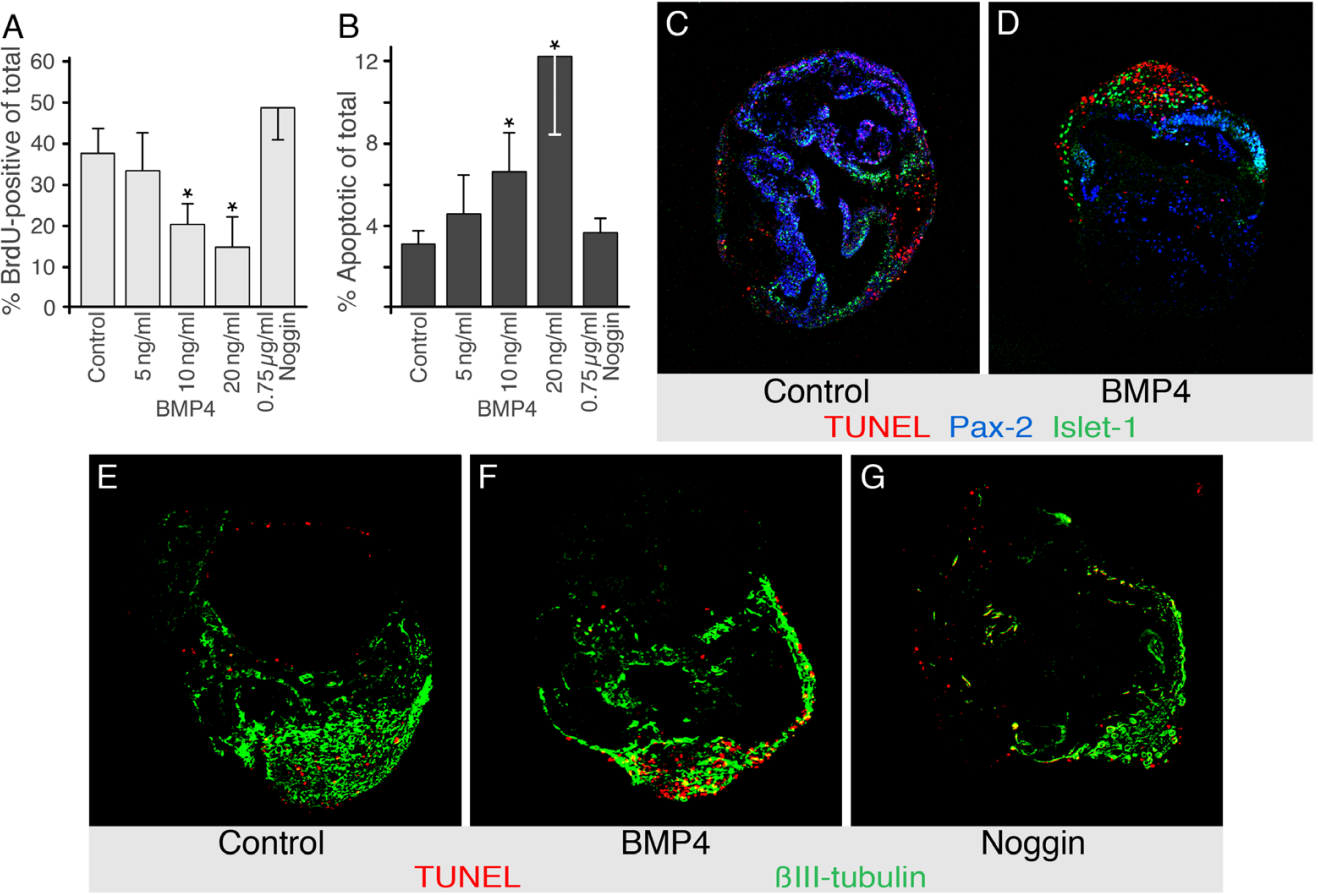
Exogenous BMP4 leads to decreased overall proliferation and increased apoptosis in the neural domain of the cultured otocysts. *(A) *BMP4, when added at 10 ng/ml or 20 ng/ml to otocysts after three days in culture, significantly inhibits proliferation, tested by an 8 h BrdU pulse 24 h after adding BMP4. (Asterisks indicate *p *< 0.05 (10 ng/ml) and *p *< 0.01 (20 ng/ml), unpaired Student's t-test, n = 7; error bars represent standard deviations). *(B) *Addition of BMP4 after three days in culture leads to an increase in TUNEL-positive cells when analyzed on the seventh day (asterisks indicate *p *< 0.01 (10 ng/ml) and *p *< 0.001 (20 ng/ml), unpaired Student's t-test, n = 7; error bars represent standard deviations). (C) In sections of control otocysts analyzed on the seventh day in culture, apoptotic cells, identified by TUNEL staining (red), mostly occurred outside of the Pax-2-positive areas (shown in blue). (D) BMP4 at 10 ng/ml noticeably increased the number of TUNEL-positive cells in the potential neural domain, identified by green islet-1-positive cell nuclei. *(E,F) *Similar to (C,D), but the neural domain is labeled with antibody to beta-III tubulin (shown in green). Note the increase of TUNEL-positive cell numbers within the neural domain. *(G) *Noggin does not affect the number of apoptotic cells.

To elucidate the effect of BMP4 on the increased generation of hair cells, we analyzed cell proliferation and apoptosis in cultures treated with exogenous BMP4 from day three in culture onward. We found that proliferation was significantly reduced in otocysts treated with BMP4 whereas apoptosis was increased (Fig. 3A, B). BMP4-induced apoptosis almost exclusively occurred in areas that we identified as neuronal using the strong islet-1 immunostaining of cochleovestibular neuron nuclei (Fig. 3C–D). Islet-1 is expressed in the developing inner ear in neurons as well as in the nascent sensory epithelia; neuronal islet-1 staining can easily be distinguished from islet-1-positive developing sensory epithelia by a more round shape of stained neuronal nuclei and higher intensity when compared to epithelial staining (see also [5]). The paired-box transcription factor Pax-2 is expressed in developing sensory epithelia and is down-regulated in cochleovestibular ganglion cells [17] and accordingly, neuronal domains in cultured otocysts lack Pax-2-expression (Fig. 3C–D). When BMP signaling was blocked with noggin, there was no significant increase in overall cell proliferation, but less apoptosis occurred in the neuronal area, identified by staining for β-III tubulin (Fig. 3E–G). Based on these results, we rule out that the increase in hair cell numbers in response to BMP4 is based on a higher rate of cell proliferation or lower rate of apoptosis.

### Otocyst cells fail to commit to a sensory epithelial fate in the absence of BMP signaling

**Figure 4:**
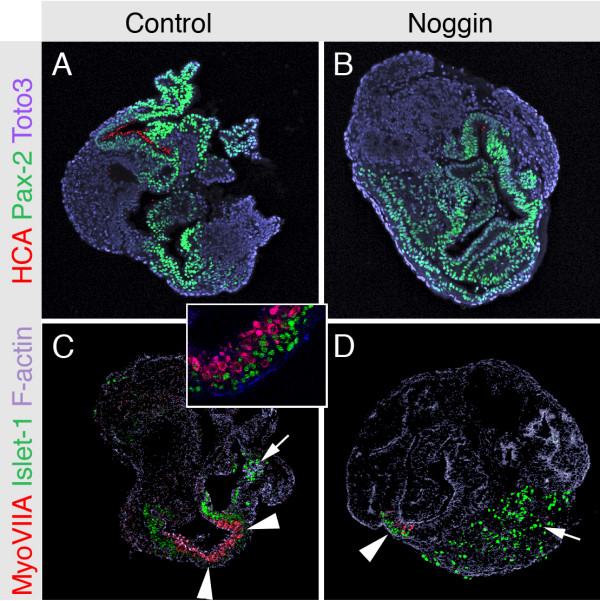
Sensory epithelium fails to develop in absence of BMP signaling. *(A,B) *Blockade of BMP signaling with 0.5 μg/ml noggin robustly decreased the number of hair cell antigen-expressing hair cells (HCA shown in red) that were detectable after seven days in culture. It appears that Pax-2 expression (shown in green) is not affected by noggin. *(C,D) *In noggin-treated otocysts, islet-1-positive sensory epithelia (visualized in green) were evidently diminished, but not completely absent (arrowheads), as shown in this representative set of images. The images shown are not representative for the neural domains where we observed that the overall number of islet-1-positive neurons (arrows in C and D) did not differ between noggin-treated and control otocysts (no significant difference, n = 5). The inset in (C) shows a higher magnification of the islet-1-positive sensory epithelia (see also Li et al., 2004b).

The sensory epithelia of the inner ear, including hair cells and supporting cells, are derived from Pax-2-positive progenitor cells that also give rise to other inner ear cell types [17-20]. The reduction in the number of hair cells in response to blockade of BMP signaling with noggin was not accompanied by a detectable overall reduction of Pax-2-positive progenitor cells (Fig. 4A, B). As the sensory epithelium progenitor cells become committed to form sensory patches, Pax-2 expression decreases and islet-1 becomes detectable in both nascent hair cells and supporting cells before the onset of expression of hair cell markers [5,17]. We found that in noggin-treated otocysts, islet-1-positive epithelial patches containing hair cells appeared noticeably diminished, whereas the more strongly islet-expressing cochleovestibular neurons were apparently not affected (Fig. 4C, D). These data support our hypothesis that the generation of sensory patches from Pax-2-expressing progenitors depends on BMP signaling.

### BMP4 inhibits cell proliferation through downregulation of Pax-2

**Figure 5:**
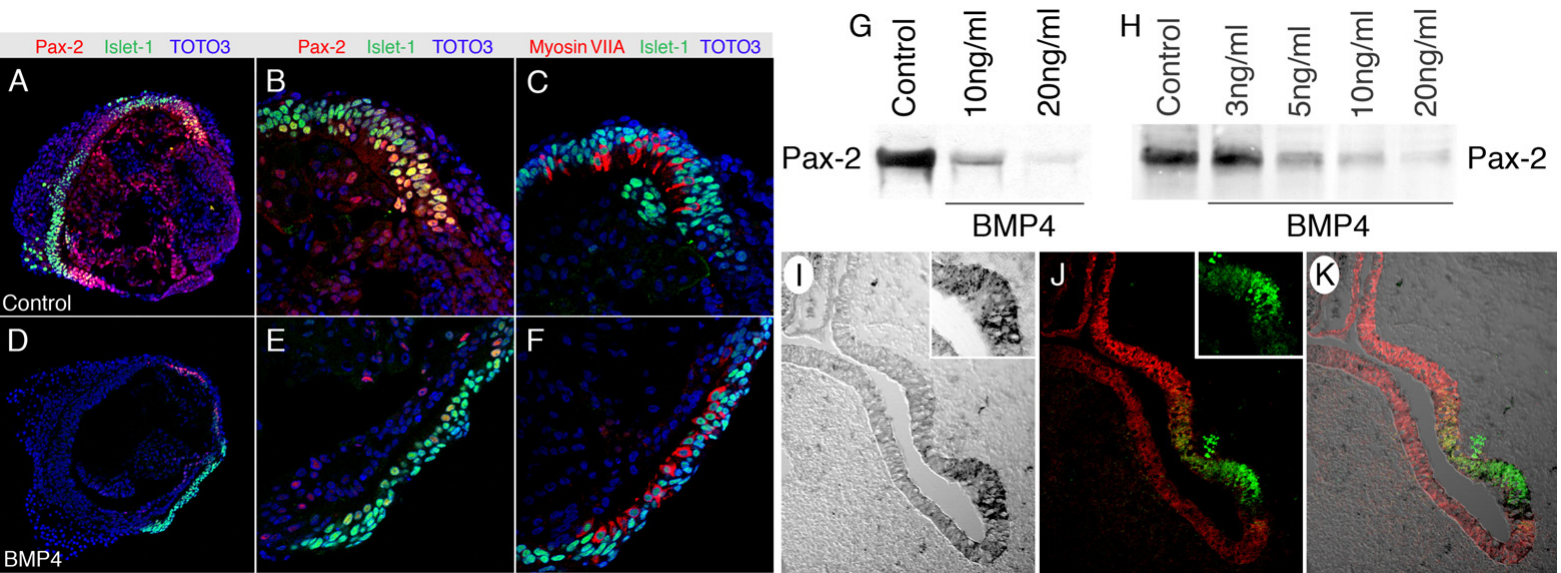
Downregulation of Pax-2 by BMP4. *(A-C) *Control group after seven days in culture. Similar to the *in vivo *situation [5], (B) islet-1-positive cells (green) appear in the incipient Pax-2-expressing sensory epithelia (red); (C) myosin VIIA-positive early hair cells (red) that express islet-1 (green) occur within islet-1-positive epithelial patches. *(D-F) *In otocysts treated with 10 ng/ml BMP4 from the beginning of the culture period, Pax-2 expression was clearly reduced, whereas islet-1-expression and hair cell generation were not affected. *(G) *In a comparative Western blot analysis, Pax-2-protein levels were clearly reduced in otocysts treated for 48 h with BMP4. The amount of protein loaded per lane was the total protein of 5 otocysts. *(H) *Similar experiment to the one shown in (G), but the duration of the BMP4-treatment was reduced to 24 h and the total protein load per lane was equalized. *(I-K) *In cryosections of stage 25 chicken inner ear, BMP4 mRNA is expressed in the basilar papilla sensory epithelium primordium (dark precipitate in (I)). (J)Pax-2 protein expression (depicted in red) becomes noticeably reduced in the BMP4-expressing area simultaneously when epithelial islet-1 expression (shown in green) occurs. Panel (K) is the merged image of (I) with (J).

The expression of Pax-2 in the early developing inner ear is usually correlated with increased cell proliferation and absence of apoptosis, which mostly occurs outside of the Pax-2-positive regions [17,21]. When BMP4 was added at the beginning of the 7d culture period, we noticed a substantial decrease in the number of Pax-2-positive cells, although without an apparent effect on the developing sensory epithelium (Fig. 5A–F). Western blot analysis corroborated that BMP4 down-regulates Pax-2 expression in a dose-dependent manner (Fig. 5G, H).

In the normal inner ear, BMP4 mRNA is used as a marker to visualize presumptive sensory epithelium [4-7]. At the crucial time during development, when sensory epithelia become postmitotic, epithelial islet-1 expression overlaps with BMP4-expressing areas (Fig. 5I–K). Conversely, in the same presumptive sensory epithelia, Pax-2 expression is downregulated (Fig. 5I–K), suggesting that BMP4 acts as suppressor of Pax-2 expression during normal inner sensory epithelium formation.

## Discussion

### BMP signaling is required for inner ear sensory organ formation

The discovery of BMP4 mRNA expression in all sensory organ primordia of the developing inner ear has led to the hypothesis that this signaling protein plays a role in the induction or differentiation of inner ear sensory epithelia [4,6]. However, because homozygous BMP4 knockout mice do not live beyond the ninth day of embryonic development it is not feasible to analyze BMP4 function during murine otogenesis.

To circumvent this problem, we used a serum-free floating culture technique, which allowed us to quantitatively test the function of BMP4 on avian inner ear sensory epithelium formation in a controlled environment. This culture regimen enabled us to quantitatively analyze the generation of inner ear cell types by using cell-specific markers. We found that the number of hair cells that formed in otocysts cultured for 7 days is considerably lower than the number of hair cells that can be found in a chicken ear at the 10^th ^day of embryonic development (E10; equivalent to E3 plus 7 days *in vitro*). Nevertheless, we argue that the chicken inner ear at E10 is also substantially larger than the 7-day cultured otocyst, which makes it difficult to assess whether a ratio of ~1.6% hair cells of the total cell number in a floating otocyst is comparable to the ratio of hair cells *versus *total inner ear cell number at E10. Nevertheless, we suggest that that the effects we observed *in vitro *are at least partially representative of the hair cell populations found in the various hair cell-bearing organs of the developing chicken ear. To the best of our ability, we removed periotic mesenchyme from the E3 otocysts. Specimens with obvious mesenchymal contribution were not scored in this study. Nevertheless, we did not observe a noticeable difference in the number of hair cells and neurons detectable in otocysts with clear mesenchymal contribution, which is an indication that the periotic mesenchyme appears to exert little influence on the otocyst after stage 16 (E3).

Loss of BMP signaling by application of noggin or a combination of dominant-negative BMP receptors markedly reduced the number of hair cells in the developing otocyst, which strongly supports the hypothesis that BMP signaling is required for hair cell generation. Nevertheless, even at the highest concentrations of noggin or dominant-negative BMP receptors, no complete blockade of hair cell generation was achieved. We suspect that the nature of BMP signaling in the developing sensory epithelia does not allow complete blockade with application of soluble inhibitors. BMP4 is a secreted protein that binds to cell surface receptors to activate the Smad signaling pathway (reviewed in [22,23]). Secreted BMP4 acts on neighboring cells in a paracrine manner as well as in autocrine fashion on the cells that produce the factor if these cells have appropriate BMP receptors. We hypothesize that extracellular BMP inhibitors cannot completely interfere with autocrine signaling, resulting in an incomplete blockade of BMP signaling in otocyst cells that express both BMP4 and BMP receptors.

Interference with BMP signaling in the inner ear *in vivo *by application of noggin or noggin-producing cells [9,10] led to inhibition or malformation of semicircular canals accompanied by occasional deformation of ampullae. No effects on sensory epithelium formation and hair cells were reported; however, these studies did not explicitly quantify the number of hair cells. Alternatively, we hypothesize that dorso-lateral transplantation of beads soaked with noggin or of noggin-producing cells into the periotic mesenchyme apparently did not suffice to reach sufficient concentrations of the inhibitor over the course of several days in the ventro-medial region of the otocyst, where the sensory epithelia originate. Using a serum-free floating culture environment allowed us to control the concentration of BMP4 or BMP signaling inhibitors more precisely than previous *in ovo *experiments.

### BMP4 promotes hair cell differentiation

Three mechanisms could account for increase of the number of hair cells by exogenous BMP4: 1) promotion of sensory epithelial progenitor cell proliferation, 2) prevention of progenitor cell apoptosis, or 3) promotion of progenitor cell differentiation to hair cells. Our results suggest that BMP4 inhibits otocyst cell proliferation, but affects apoptosis only in the neuronal domain of cultured otocycts, implying that a plausible mechanism of BMP4's effect on increasing the number of hair cells is to promote sensory epithelium progenitor cells to differentiate.

Another line of evidence for BMP4's effect on sensory epithelium progenitor cells arises from our loss-of-function analysis with noggin. Here we found that noggin-treatment did not affect the number of Pax-2-positive cells, whereas the number of hair cells was considerably reduced. This result implies that Pax-2-positive progenitors fail to differentiate into sensory epithelium when BMP signaling is blocked.

Our observations that BMP4-treatment leads to increased hair cell generation and to reduction of otocysts cell proliferation, suggests a dual, concentration-dependant, action of BMP4: at concentrations of 3–5 ng/ml BMP4 augments cell differentiation. In fact, we did not find a significant inhibition of cell proliferation with BMP4 concentrations below 10 ng/ml (data not shown). At higher BMP4 concentrations (10–20 ng/ml), cell proliferation is sufficiently reduced to offset the BMP4 effect on hair cell differentiation. The result of the concentration-dependant dual effects of BMP4 is manifested in a bell-shaped dose-dependency of hair cell numbers (Fig. 2E).

The effects of BMP4 on otocyst cell proliferation and apoptosis that we report here differ from, but do not contradict, results obtained by *in vivo *administration of noggin to the developing chicken inner ear [9,10]. In these experiments, cell proliferation decreased and apoptosis increased after noggin-secreting beads or cells were transplanted into the mesenchyme adjacent to the otocysts. We argue that these previous analyses and our experimental protocol are not easily comparable as many factors could account for the observed differences. For example, interactions of noggin with potential signals in the periotic mesenchyme might produce different effects on inner ear tissue when compared with mesenchyme-free otocyst cultures. Another possible explanation is that our analysis included all otocyst-derived cells, whereas previous studies focused on the parts of the developing inner ear surrounding the noggin-secreting grafts.

The effect of BMP4 on promoting differentiation of sensory epithelium progenitor cells is consistent with its temporal and spatial expression in the inner ear sensory organ development [4,6,7]. BMP4 mRNA is expressed in all sensory organ primordia before the hair cells and supporting cells are developed. At this crucial period of sensory organ formation, the progenitor cells downregulate Pax-2, leave the cell cycle, and initiate differentiation into hair cells and supporting cells; Pax-2 expression subsequently persists in hair cells [5,17]. These events appear to be correlated with the presence of BMP4 in sensory patches, supporting a possible role for BMP4 in controlling proliferation and specifying differentiation in the inner ear's sensory epithelia.

Additional clues about the potential targets of BMP4 in the developing inner ear could arise from analysis of expression of BMP receptors and components of the BMP signaling pathways. Beside a comprehensive study in the developing zebrafish ear and lateral line [24], only one study has addressed in the developing chicken inner ear expression of BMP receptors and Smad proteins, the intracellular transducers of BMP signaling [25]. Although the latter study was focused on the role of BMP signaling during development of semicircular canals, it should be noted that the expression of BMP receptors in the developing chicken inner ear is probably not restricted to sensory patches or the Pax-2-positive domain. A more widespread expression of BMP receptors could potentially be the reason for the substantial downregulation of Pax-2 protein that we observed in free-floating otocycts in response to BMP4 treatment. At least in the developing zebrafish inner ear several BMP receptors are expressed widely, some of them ubiquitously, at all developmental stages [24].

### BMP4 is a crucial control factor of proliferation, differentiation and apoptosis in the developing inner ear

At early stages of inner ear development, all cells of the otic placode and the otic pit express the transcriptional regulator Pax-2. Subsequently and noticeably evident at embryonic day three, Pax-2-expression gets more localized to the medio-ventral part of the otic vesicle, the region where the sensory epithelia originate [18-20]. At this time of development, cell proliferation mostly occurs in the Pax-2 expression domain and apoptosis happens outside of the Pax-2 positive domain [17]. We show that the numbers of Pax-2-positive cells as well as proliferating cells are markedly reduced when exogenous BMP4 is added to cultured otocysts. Coinciding with decreasing numbers of Pax-2-positive cells, we also detected substantial downregulation of the expression of Pax-2 protein. In the developing sensory patches, we were able to visualize that BMP4 mRNA is detectable in the incipient sensory patches just at the time when islet-1 expression becomes evident. The onset of islet-1 expression in sensory patches coincides with early differentiation of sensory epithelial cells [5]. In parallel, Pax-2 protein is markedly downregulated in these patches, implying that BMP4 also causes downregulation of Pax-2, cessation of proliferation, and upregulation of markers for early differentiating sensory epithelium *in vivo*.

## Conclusion

In summary, our results indicate that BMP signaling is involved in the generation of inner ear sensory patches and hair cells. We also found evidence that BMP4 can affect cell proliferation and apoptosis in different cell populations of the developing inner ear. We therefore conclude that correct control of BMP signaling in all areas of the developing otocyst is central to obtaining an accurate balance of cell proliferation, apoptosis and cell differentiation needed for correct morphogenesis of the inner ear's sensory organs.

## Methods

### Chicken embryos

Fertilized chicken eggs of the white leghorn strain (Charles River SPAFAS) were stored at 14°C until embryonic development was initiated by placing them onto rocking platforms into a humidified incubator maintained at 38°C. Embryos were staged according to Hamburger and Hamilton [26].

### Otocyst culture

Otocysts from stage 15–16 embryos were dissected in phosphate-buffered saline (PBS, pH7.2) and incubated for 30s in trypsin (0.125% in PBS) to aid in the removal of the periotic mesenchyme. The otocysts were rapidly transferred into 10 mL of serum-free culture medium in Petri dishes (non-tissue culture treated). Culture medium was mixed from equal parts of high glucose Dulbecco's modified Eagle's medium and F12 medium supplemented with N2 and B27 (Media and supplements were from Invitrogen/GIBCO/BRL, Carlsbad, CA). BMP4, noggin, and soluble BMPR1a and BMPR1b were obtained from R&D Systems (Minneapolis, MN). BMP signaling antagonists were continuously supplemented throughout the culture period. BMP4 was either applied from the beginning of the culture period or from the third day (72 h) in culture onward. The floating otocysts were maintained in a humidified incubator in a 5% CO_2 _atmosphere at 37°C. For quantitative analysis, cultured otocysts were harvested on the seventh day in culture, fixed overnight with paraformaldehyde (4% in PBS), cryoprotected for 48 h in sucrose (30% in PBS), embedded in TissueTek (EMS) and serially sectioned (16 μm thick) with a cryostat (CM3050, Leica).

### 5-bromo-2-deoxyuridine (BrdU) labeling

BrdU (Sigma) was added (3 μg/ml) at specific time points for an exposure period of 8 h, after which the otocycts were harvested and processed for cryosectioning. BrdU incorporation was detected immunohistochemically (see below). Quantification was done by determining the fraction of BrdU-positive cells of the total cell number in every other section obtained from each otocyst.

### Apoptosis labeling

Apoptotic cells were identified with the TUNEL labeling technique [27] (TUNEL enzymatic labeling kit, Roche). The fixed sections were washed three times with PBS, 5 min each, treated for 2 min with ice-cold 0.1% sodium tartrate, and incubated for 1 h with fluorescein-dNTP and terminal deoxynucleotideyl transferase at 37°C. After triple washing with PBS, the sections were immunostained for additional cell markers (see below).

### Immunohistochemistry

The cryosections were blocked for 1 h with 1% BSA, 5% heat-inactivated goat serum and 0.1% Triton-100 in PBS (PBT1). For BrdU labeling, the sections were exposed to 2N HCl for 30 min before adding the primary antibody. The slides were then incubated overnight at 4°C in PBT1 with diluted antibodies: 1:5000 for mouse anti-HCA (a gift from G. Richardson [28]), 1:3000 for rabbit anti-myosin VIIA (a gift from A. El-Amraoui and C. Petit), 1:100 for monoclonal anti-islet-1 (clone 40.3A4, cell culture supernatant, Developmental Studies Hybridoma Bank, University of Iowa, Iowa City, IA), 1:100 for rabbit anti-Pax2 (Covance, Princeton, NJ), 1:500 for monoclonal anti-neuron-specific β-III tubulin (TuJ) (Chemicon), and 1:1000 for mouse anti-BrdU (Sigma). Unbound antibodies were removed with three PBT1 washes and one PBT2 (same as PBT1 but without serum) wash for 15 min each at room temperature. FITC-conjugated, TRITC-conjugated, and cy5-conjugated goat anti-rabbit and anti-mouse secondary antibodies (Jackson ImmunoResearch) were used at a dilution of 1:400 in PBS. A 45–60 min incubation period in the secondary-antibody mixture preceded three washes for 15 min each in PBS. Counterstaining with long-wavelength nuclear staining agent TOTO-3 (Molecular Probes) was done to visualize cell nuclei and TRITC-conjugated phalloidin was used to visualize filamentous actin. The coverslipped slides were analyzed by confocal microscopy (TCS SP2, Leica). For quantitative studies, we used the data measured in all serial sections of individual specimen and we calculated mean values from individual specimens derived from at least three independent experiments. For counting of labeled cell nuclei, we analyzed each section with ImageJ (version 1.31 v for Mac OSX available at ). A section thickness of 16 μm cannot completely exclude double counting of hair cells but assessment of pilot experiments with complete series' of sections counterstained with TOTO-3 did not reveal any evidence that our analysis overestimated hair cell numbers. Incomplete series were not used for quantitative assessments of cell numbers.

### *In situ *hybridization/fluorescent immunostaining

Digoxigenin-labeled sense and antisense probes for chicken BMP4 were synthesized (DIG RNA Labeling Kit, Roche) from 1 μg of linearized plasmid DNA and resuspended in 100 μl water. *In situ *hybridization was initiated by rehydrating the sections in 100 μl diluted probe (1:100) in 50% (v/v) formamide, 10% (w/v) dextran sulfate, 1 mg·ml^-1 ^yeast RNA, 1 × Denhardt's solution, 185 mM NaCl, 5.6 mM NaH_2_PO_4_, 5 mM Na_2_HPO_4_, 5 mM EDTA, and 15 mM Tris at pH7.5 and preheated to 70°C. After coverslipping and overnight incubation at 65°C in a chamber humidified with 50% (v/v) formamide in 150 mM NaCl and 15 mM trisodium citrate at pH7 (1 × SSC), the coverslips were removed in 5 × SSC and the slides were washed twice for 30 min each in 50% (v/v) formamide and 0.1% (v/v) polyoxyethylene sorbitan monolaurate (Tween-20) in 1 × SSC at 65°C. Thereafter, the slides were washed for 15 min in 0.2 × SSC and for 15 min in PBS at room temperature.

For the first antibody detection, the sections were blocked for 1 h in PBT1. The slides were then incubated for 2 h at room temperature with alkaline phosphatase-conjugated anti-digoxigenin Fab fragments in PBT1 (1:1000; Boehringer Mannheim). Unbound Fab fragments were removed by washing twice for 30 min each in PBT2. For detection, the sections were covered with 100 μl of nitro blue tetrazolium chloride and 5-bromo-4-chloro-3-indolyl phosphate substrate (1-STEP NBT/BCIP, Pierce), coverslipped, and incubated overnight at room temperature in a humidified chamber. For the second immunostaining with fluorescent antibodies, coverlips were removed in PBS and the slides were incubated overnight at 4°C in PBT1 with primary antibodies to Pax-2 and islet-1. Fluorescent secondary antibody detection and image acquisition by confocal microscopy was conducted as described above.

### Western blot analysis

Proteins were separated on 7.5% SDS-polyacrylamide gels and transferred to nitrocellulose membranes using a semi-dry transfer system (Trans-Blot SD, BioRad). Relative amounts of total protein and concentration differences among samples were determined with a protein assay kit (BCA kit, PIERCE). Western blot membranes were incubated for 1 h at room temperature in 2.5% (vol/vol) Liquid Block (Amersham Pharmacia Biotech) and 0.1% (vol/vol) Tween-20 in PBS. Pax-2 antiserum was diluted 1:250 in 2.5% (vol/vol) Liquid Block and 0.1% (vol/vol) Tween-20 in PBS and blots were incubated overnight at 4°C in diluted antiserum. Unbound primary antibodies were removed by four washes for 15 min each at room temperature in 0.1% Tween-20 in PBS. Bound primary antibodies were detected with horseradish peroxidase-conjugated antibody to rabbit IgG (Amersham Pharmacia Biotech) at a dilution of 1:7500 in 0.1% Tween-20 in PBS. Unbound secondary antibodies were removed by two washes of 15 min each in 0.1% Tween-20 in PBS and two washes for 15 min each in PBS. Detection was performed with chemiluminescence substrate (ECL plus, Amersham Pharmacia Biotech) and exposure to Hyperfilm ECL (Amersham Pharmacia Biotech).

## Authors' contributions

HuL carried out most otic vesicle culture experiments, immunohistochemistry, in situ hybridization, microscopical imaging, and statistical analysis. EC participated in immunohistochemical and quantitative analyses and performed Western blot experiments. ZW, YZ, and YW provided corroborating evidence for the effects observed and contributed additional quantitative data. HoL carried out cryosectioning and organotypic culture experiments. HuL and SH conceived of the study, HuL drafted the manuscript and SH finalized the manuscript, which has been read and approved by all authors.
